# MetaPrism: A versatile toolkit for joint taxa/gene analysis of metagenomic sequencing data

**DOI:** 10.1093/g3journal/jkab046

**Published:** 2021-03-13

**Authors:** Jiwoong Kim, Shuang Jiang, Yiqing Wang, Guanghua Xiao, Yang Xie, Dajiang J Liu, Qiwei Li, Andrew Koh, Xiaowei Zhan

**Affiliations:** 1 Quantitative Biomedical Research Center, Department of Population and Data Sciences, University of Texas Southwestern Medical Center, Dallas, TX, 75390, USA; 2 Department of Statistical Science, Southern Methodist University, Dallas, TX 75275, USA; 3 Harold C. Simmons Cancer Center, University of Texas Southwestern Medical Center, Dallas, TX 75390, USA; 4 Department of Bioinformatics, University of Texas Southwestern Medical Center, Dallas, TX 75390, USA; 5 Department of Public Health Sciences, Pennsylvania State University, Hershey, PA, 17033, USA; 6 Department of Mathematical Sciences, The University of Texas at Dallas, Richardson, TX 75080, USA; 7 Department of Microbiology, University of Texas Southwestern Medical Center, Dallas, TX, 75390, USA; 8 Department of Pediatrics, University of Texas Southwestern Medical Center, Dallas, TX 75390, USA; 9 Center for Genetics of Host Defense, University of Texas Southwestern Medical Center, Dallas, TX 75390, USA

**Keywords:** metagenomics sequence analysis, joint analysis, microbiome biomarker

## Abstract

In microbiome research, metagenomic sequencing generates enormous amounts of data. These data are typically classified into taxa for taxonomy analysis, or into genes for functional analysis. However, a joint analysis where the reads are classified into taxa-specific genes is often overlooked. To enable the analysis of this biologically meaningful feature, we developed a novel bioinformatic toolkit, MetaPrism, which can analyze sequence reads for a set of joint taxa/gene analyses to: 1) classify sequence reads and estimate the abundances for taxa-specific genes; 2) tabularize and visualize taxa-specific gene abundances; 3) compare the abundances between groups; and 4) build prediction models for clinical outcome. We illustrated these functions using a published microbiome metagenomics dataset from patients treated with immune checkpoint inhibitor therapy and showed the joint features can serve as potential biomarkers to predict therapeutic responses. MetaPrism is a toolkit for joint taxa and gene analysis. It offers biological insights on the taxa-specific genes on top of the taxa-alone or gene-alone analysis.

MetaPrism is open-source software and freely available at https://github.com/jiwoongbio/MetaPrism. The example script to reproduce the manuscript is also provided in the above code repository.

## Introduction

The human microbiome consists of ∼39 trillion bacteria and influences host health (Sender *et al*. 2016). Recently, the use of metagenomic sequencing has become increasingly popular as a more unbiased approach to gut microbiome profiling as compared to 16S rRNA sequencing. A common approach to comparing differences in the gut microbiome between groups (cases and controls) is to identify significant differences in either taxa or microbial genes. Several popular bioinformatic tools have been developed for this purpose, including MetaPhlAn2 (Truong *et al*. 2015), Kraken (Wood and Salzberg 2014), HUMAnN2 (Franzosa *et al*. 2018), and FMAP (Kim *et al*. 2016b) (Table S1). However, these tools analyze either taxonomic abundances (taxonomic profiling) or gene abundances (function profiling) separately. As each microorganism carries its own genes, taxonomic and functional profiling results are not intrinsically independent. In fact, recent discoveries demonstrated that taxon-specific genes have a causative role in disease progression and treatment responses. For example, Duan et al. found that a specific *Enterococcus faeclis* carrying the cytolysin gene promotes alcoholic liver disease (Duan *et al*. 2019). Simms-Waldrip et al. found that the antibiotic resistance genes in the graft-versus-host-disease patients are enriched for *Klebsiella* (Simms-Waldrip *et al*. 2017). Therefore, a joint analysis, where taxonomy and functional features are analyzed together, could provide useful biological and clinical insights (Langille 2018). However, bioinformatics tools for joint analyses are lacking.

Our innovation in this manuscript is to define and utilize joint taxa/gene features via bioinformatics approach, with the goal of offering biologically interpretable findings. For example, our method characterizes the genes discovered for each species. This facilitates quantitative analysis of gene abundances in a species-specific manner, which is usually not readily available. Our approach is initiated from *de novo* assembled contigs, which are both taxonomically and functionally annotated. Our simulations showed this method could accurately detect bacterial species and their carried genes. In a recent review article (Langille 2018), Langille prompted that understanding the gene contents at species level can offer better interpretation than using the taxon or gene content alone, and potentially improve outcome predictions. This confirmed that the joint feature is useful for general microbiome studies. Our tool provided these joint features as the first step for a wide range of downstream analysis tasks. For example, we demonstrated that the quantity of taxa-specific gene abundances is a potentially useful biomarker to predict the immunotherapy responses.

To facilitate joint analysis, we developed MetaPrism, a novel bioinformatics tool to (1) classify metagenomic sequence reads into both taxa and gene level, (2) normalize the taxa-specific gene abundances within samples, (3) tabularize or visualize these joint features, (4) perform comparative microbiome studies, and (5) build prediction models for clinical outcomes. Using simulated sequence reads, we validated that the performance of MetaPrism is accurate. We further applied the MetaPrism analysis to an immune checkpoint therapy and detected novel joint features as potential biomarkers.

MetaPrism is open-sourced and is available at https://github.com/jiwoongbio/MetaPrism. Given the advantages of joint analysis, MetaPrism is a useful tool for a wide range of microbiome-metagenomic sequence studies.

## Materials and Methods

### Analysis workflow

**Figure 1.**
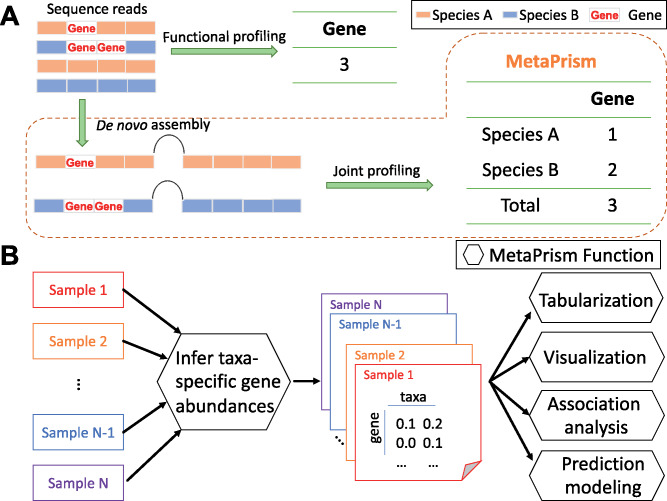
**A schematic illustration of the algorithm and the functions in MetaPrism. A)** Illustration of the MetaPrism algorithm to infer taxa-specific gene abundances. While function profiling infers that three reads are mapped to a gene, it cannot provide further taxonomic information. Through joint profiling, MetaPrism can utilize *de novo* assembled contigs to estimate taxa-specific features: two gene copies are from species A and one copy is from species B; **B)** An overview of the joint analysis workflow in MetaPrism. The hexagon shapes represent implemented functions in MetaPrism.

MetaPrism is a toolkit for joint analysis tasks. At its core, MetaPrism will infer the taxa and gene for each metagenome sequence read. One approach is to align each read to bacterial nucleotide reference genomes to obtain its taxonomy and align it to a protein database to obtain its gene functions. However, this approach is technically challenging: due to the short lengths of Illumina sequence reads and the high sequence similarities between bacteria genomes, alignment of short reads is not feasible. We thus developed a novel algorithm (Figure 1A) in an integrated toolkit (Figure 1B) to tackle this challenge.

First, we perform *de novo* assembly for each sample using metaSPAdes (Nurk et al. 2017) with all metagenomic sequence reads to obtain long contigs. These contigs are much longer than sequence reads, which allows for accurate taxonomical and functional profiling.

Second, we identify the taxonomy of these contigs. All the contigs are aligned to a large reference database of more than 4,000 bacterial genomes using centrifuge (Kim *et al*. 2016a). Ambiguous alignments will be filtered out from the subsequent analysis.

Third, we identify genes and their locations from the contigs. We detect the open reading frames from the contigs, translate the nucleotide bases to amino acids, and align them using DIAMOND (Buchfink *et al*. 2015) to a protein database. To comprehensively investigate all bacteria genes, either KEGG protein databases that include protein sequences from KEGG orthologue genes (Kanehisa *et al*. 2012) or KFU (KEGG orthology with UniProt protein sequences) (Kim *et al*. 2016b), can be utilized. By default, we require minimum coverage of 0.8 to ensure good protein alignments.

Lastly, we calculate and normalize gene abundance within-sample. We align metagenomic sequence reads to the contigs using BWA (Li and Durbin), and count the number of aligned reads located in the genes of interest. We calculate the read depth normalized by contig length, and this quantity is denoted as mean depth to represent the gene abundances. Larger numbers often indicate higher gene abundance. Other abundance statistics, such as FPKM (Fragment Per Kilobase of transcript per Million reads) or depth per genome (normalized read depth per taxa genome length), are also provided.

Through the above steps, we obtain the joint feature where the gene abundances are associated with taxonomy information. These features can be viewed as novel microbiome measurements, which provide more information that taxonomic abundances or gene abundances alone. To utilize these features, MetaPrism provides four downstream analysis modules (Figure 1B): (1) tabularization module allows to export the joint features such as the mean depth of genes per contig at the genus level; (2) visualization module allows to visualize the abundances of the joint features in an HTML webpage; (3) differential abundances analysis modules can calculate the fold change of the gene abundances for two groups of samples along with statistical significances in terms of p-values; and (4) prediction module can construct a random forest model or extreme gradient boosting model to detect joint abundance features as potential biomarkers (Breiman 2001; Chen and Guestrin 2016). In all, these modules provide a common set of functions for the typical analysis of joint features. Meanwhile, users are in full control to utilize the exported tabular data for their customized analysis. A list of available functions, command line, and major customization options in MetaPrism are listed in Table S2.

### Simulation setting

To assess the accuracy of the joint features estimated by MetaPrism, we conducted a simulation study using simulated sequence reads from a collection of bacteria species. First, we selected all 118 bacterial species with complete reference genomes where the latest genome collected from June 8, 2018 (Table S3). Then, we downloaded their sequences from NCBI FTP (http://ftp://ftp.ncbi.nlm.nih.gov/genomes/genbank/bacteria). These sequences include 229 contigs including both bacterial chromosomes and plasmids. Their lengths range from 1,308 bp to 10,236,715 bp (mean length is 1,969,971 bp). Finally, we simulated shotgun metagenomic sequencing reads and generated at 10X coverage to resemble typical read lengths from the Illumina using ART (Huang *et al*. 2012). Specifically, we set read length to be 100 base pair and the mean and standard deviation of the fragment size to be 200 bp and 50 bp, respectively.

### Data analysis for the microbiome in an immune checkpoint therapy

Immune checkpoint therapy is a revolutionary cancer treatment regime. Researchers realize that the gut microbiome plays an indispensable role in modulating the immune system and boost the therapy efficacy (Frankel *et al*. 2017). We demonstrated a joint analysis using MetaPrism to build a therapy-response prediction model. We collected stool samples of 12 melanoma patients before anti-PD1 (pembrolizumab) therapy and performed metagenomic sequencing (Frankel *et al*. 2017). Six patients responded to the therapy and six did not. We performed quality-control procedures on the metagenomic sequence reads. That included the removal of human contamination as previously described (Frankel *et al*. 2017).

## Results

### Joint features inferred by MetaPrism are accurate in simulation

We evaluate the gene abundances calculated by MetaPrism and other methods to the true abundances. We determined the true abundances by the multiplication of sequence depth and the depth of KEGG ortholog (KO) genes in the reference genomes. Notably, there could be more than one copy of KO genes in one contig; thus, the true abundance of KO genes can vary from 0 to 1,200. This is also verified by aligning the gene sequences to the KEGG protein database using DIAMOND (Buchfink *et al*. 2015).

**Figure 2.**
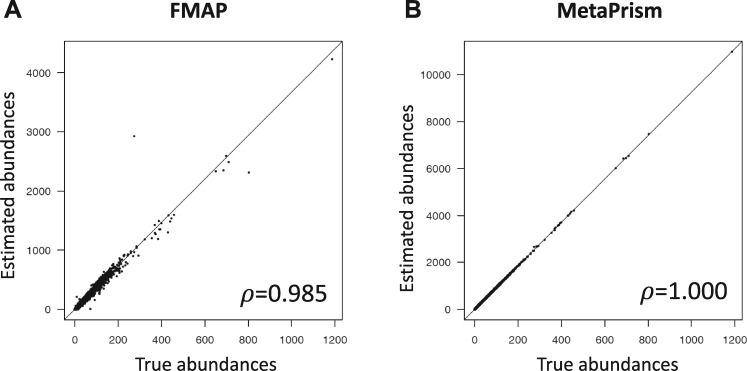
**Comparison of gene abundances reported by FMAP and MetaPrism.** We used simulations to compare the estimated gene abundances using FMAP and MetaPrism. The Pearson correlation coefficients between true abundances and the software-estimated abundances were listed on the bottom right.

We use the simulated reads totally $4.2\times 10^{9}$ nucleotide bases. We ran two programs: MetaPrism and FMAP. The FMAP software used translation alignment (BLASTX) and our previous benchmarks showed it can report gene abundances accurately (Kim *et al*. 2016b). Another popular approach is HUMAnN2. However, our simulation showed that its performance to report KO gene abundances is not accurate (**Supplementary: Simulation results using HUMAnN2**). In Figure 2, we visualized the true abundances (X-axis) and the estimated abundances (Y-axis) for FMAP and MetaPrism using scatterplots. The correlation coefficient (ρ=1.000) from MetaPrism is higher than that from FMAP (ρ=0.985). In brief, this simulation mimics a metagenomic sequence data from known species. We inferred the gene abundances using FMAP (Kim, *et al*., 2016b) and MetaPrism, and the benchmark showed that gene abundances inferred by MetaPrism were accurate and achieved the highest correlation between inferred abundances and true abundances (Figure 2**)**.

### Joint features can be potential biomarkers in immune checkpoint therapy

**Figure 3.**
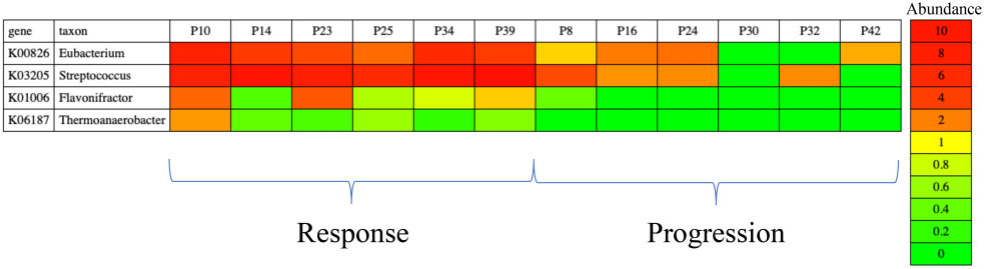
**Heatmap of joint features for predicting immune checkpoint therapy response.** We used MetaPrism_heatmap.pl to visualize four joint features (taxa-specific gene abundances, with variable importance values greater than 50%) in the immune checkpoint therapy study. The colors from red to green represent the increased gene abundances, the mean depth normalized by the contig lengths. P10, P14, P23, P25, P34, and P39 are patients who respond to the therapy; P8, P16, P24, P30, P32, and P42 are patients having progressive outcomes. K00826, branched-chain amino acid aminotransferase; K03205, type IV secretion system protein VirD4; K01006, pyruvate, orthophosphate dikinase; K06187, recombination protein RecR.

We used MetaPrism on the remaining sequence reads (detailed data retrieval, analysis steps, and command lines were available in **Supplementary: Discover species-specific biomarker in an immune checkpoint therapy study**). On average, each sample has 1.2 billion reads. We profiled sequence in MetaPrism and there are on average 24,532 joint features consisting of 2,058 taxa and 3,432 KO genes per sample. Next, we used MetaPrism to normalize read counts for each sample by reporting the mean depth per assembled contig. As demonstrated in previous simulation, the inferred abundance represents the gene counts of specific taxa. These taxa-specific gene abundances were ranked using a random forest model with 500 trees and leave-one-out cross-validation. This prediction model reached 69% accuracy to predict the immunotherapy responses. It was higher than the accuracy using taxa features alone (54%), gene features alone (62%), or just random guess (50%). The prediction accuracy based on the proposed joint features achieved a 7% lead compared to the second-best model where gene features were used. Furthermore, it detected four joint features with variable importance greater than 50%. We examined the abundances these abundances with red to green colors representing the depth values (Figure 3). We observed these joint features are more abundant in the responder group suggesting that they may improve the treatment efficacy. Among them, the most important feature is the K00826 gene (branched-chain amino acid aminotransferase, *BCAT1*) from the genus *Eubacterium* (Table 1). The average abundance of this joint feature in the response group is three-fold higher compared to that in the progression group (response = 4.70 vs progression = 1.17). Interestingly, BCAT1, as an important enzyme in branched-chain amino acid, is associated with glycolysis and oxygen consumption (Kelly and Pearce 2020). These biological procedures determine the cancer growth (Bertout *et al*. 2008; Yttersian Sletta *et al*. 2017), and they may be interfered by the high activity of BCAT of *Eubacterium*, the top abundant taxon in this dataset (20.9%). Although alteration of BCAA metabolism from the bacterial contributes to creating a tumor-favoring metabolic condition in the host remains a hypothesis, further mechanistic studies may investigate the K00826 genes from *Eubacterium* as a biomarker for cancer immune checkpoint therapy.

**Table 1.** **Prediction models and performances for taxonomical analysis, functional analysis, and joint analysis.** We tabularized the details of prediction models used in three types of analyses and their prediction performances.

	Taxonomic profiling	Functional profiling	Joint profiling
**Model**	Random forest	Random forest	Random forest
**Number of trees**	500	500	500
**Number of features**	1,048	5,227	62,086
**Top features (if variable importance > 50%) ^#^**			
**1^st^ feature**	Chondromyces (100)	K07705 (100)	K00826 *Eubacterium* (100)
**2^nd^ feature**	Roseateles (65)	–	K03205 *Streptococcus* (89)
**3^rd^ feature**	–	–	K01006 *Flavonifractor* (81)
**4^th^ feature**	–	–	K06187 *Thermoanaerobacter* (74)
Accuracy*	53.8%	61.5%	69.2%

# : The variable importance values are listed in parentheses.

* : Prediction accuracy was evaluated using leave-one-out cross-validations.

In terms of computation, all the above analyses can be accomplished on a standard computation cluster (e.g., 128 GB memory with 2 GB hard drive space per sample).

## Discussion

We present a novel bioinformatics tool, MetaPrism. It implements functions to quantify the joint features (both taxonomic and functional) from metagenomic sequence reads, as well as other functions for downstream data analyses including comparative studies and prediction modeling. We demonstrate that the joint features can provide novel insights to understand the microbial role in a cancer immunotherapy study.

MetaPrism is flexible and can be customized. For example, we can prepare a specific gene database to investigate taxa-specific antibiotic resistance genes (ARGs). We have used reference protein databases with ARGs, such as ARDB (Liu and Pop 2009) or CARD (McArthur *et al*. 2013). In a graft-versus-host disease (GVHD) study, we used MetaPrism with the ARDB to infer taxa-specific ARGs for joint resistome profiling. Then we correlated patients’ resistome to the outcome of GVHD. We found increased abundances of antibiotic-resistance genes (e.g., *mdtG, AcrA, AcrB*, and *TolC*) in *Klebsiella* and *E. coli* in the GVHD patients compared with the abundances in non-GVHD patients. This finding may hint optimal antibiotic prescription for better management of GVHD.

MetaPrism characterizes the joint features based on the contigs that are *de novo* assembled from metagenomic sequence reads. This is a distinct feature compared with other software. For example, HUMAnN2 used a tiered search strategy that relied on a curated reference database for organism-specific genes (Franzosa *et al*. 2018). However, many bacterial genes are shared across organisms and can be missed by the organism-specific gene database. Thus, we designed the MetaPrism to reduce the dependency on curated reference databases. The tradeoff for this decision is that MetaPrism requires more computational resources for the *de novo* assembling step.

In all, MetaPrism is free and useful software to facilitate joint analyses and it is suitable for general microbiome studies. Researchers can expect MetaPrism to quantify species-specific gene abundances and use these interpretable features in association studies and prediction tasks.

## Data Availability

The metagenomic shotgun sequence dataset in the immune checkpoint therapy is available from the NCBI BioProject PRJNA397906. The treatment responses for the 12 patients as well as the analysis codes were available in the **Supplementary: Discover species-specific biomarker in an immune checkpoint therapy study**. The source codes of MetaPrism software are available at: https://github.com/jiwoongbio/MetaPrism. That resource contains the software requirements, usage example, and documentations for all MetaPrism components (e.g., download bacterial database, quantify species-specific gene abundances, build association models and prediction models, tabularize results, and visualize results in heatmap plots). Supplemental material is available at figshare DOI: https://doi.org/10.25387/g3.13944521.
